# Markerless Mouse Tracking for Social Experiments

**DOI:** 10.1523/ENEURO.0154-22.2023

**Published:** 2024-02-23

**Authors:** Van Anh Le, Toni-Lee Sterley, Ning Cheng, Jaideep S. Bains, Kartikeya Murari

**Affiliations:** ^1^Electrical and Software Engineering, University of Calgary, Calgary, AB T2N 1N4, Canada; ^2^Hotchkiss Brain Institute, University of Calgary, Calgary, AB T2N 1N4, Canada; ^3^Faculty of Veterinary Medicine, University of Calgary, Calgary, AB T2N 1N4, Canada; ^4^Alberta Children’s Hospital Research Institute, University of Calgary, Calgary, AB T2N 1N4, Canada; ^5^Biomedical Engineering, University of Calgary, Calgary, AB T2N 1N4, Canada

**Keywords:** computer vision, deep learning, mouse tracking, social behavior

## Abstract

Automated behavior quantification in socially interacting animals requires accurate tracking. While many methods have been very successful and highly generalizable to different settings, issues of mistaken identities and lost information on key anatomical features are common, although they can be alleviated by increased human effort in training or post-processing. We propose a markerless video-based tool to simultaneously track two interacting mice of the same appearance in controlled settings for quantifying behaviors such as different types of sniffing, touching, and locomotion to improve tracking accuracy under these settings without increased human effort. It incorporates conventional handcrafted tracking and deep-learning-based techniques. The tool is trained on a small number of manually annotated images from a basic experimental setup and outputs body masks and coordinates of the snout and tail-base for each mouse. The method was tested on several commonly used experimental conditions including bedding in the cage and fiberoptic or headstage implants on the mice. Results obtained without any human corrections after the automated analysis showed a near elimination of identities switches and a ∼15% improvement in tracking accuracy over pure deep-learning-based pose estimation tracking approaches. Our approach can be optionally ensembled with such techniques for further improvement. Finally, we demonstrated an application of this approach in studies of social behavior of mice by quantifying and comparing interactions between pairs of mice in which some lack olfaction. Together, these results suggest that our approach could be valuable for studying group behaviors in rodents, such as social interactions.

## Significance Statement

There is an increasing need for tools that accurately track animals during social interactions. Currently, state-of-the-art deep-learning-based approaches are highly successful and generalizable to different situations, but commonly require significant human effort in iterative training and refinement to maintain identity assignment of individuals during interactions. Here, we present a new approach that reliably tracks two mice within a constrained set of experimental conditions: a top-down camera view with controlled illumination, bedding in cage and fiberoptic, and/or headstage implants. Using this approach, we report a near elimination of identities switches and a ∼15% improvement in tracking accuracy over pure deep-learning-based keypoint tracking approaches trained on the same data and without any human correction, albeit within our chosen set of experimental conditions.

## Introduction

Social interactions are a cornerstone of human society. Social deficits are a hallmark of several neurodevelopmental disorders such as autism spectrum disorders (DiCicco-Bloom et al., 2006) and attention deficit hyperactivity disorder (Harpin et al., 2016). Animal models, particularly mouse models, are widely used to investigate social behavior and the mechanisms of such diseases. Measurements of social interactions in mice under normal, diseased, and treatment conditions are important components of such studies. The assessment of social behavior, commonly conducted by human annotators, can be subjective, time-consuming, and labor-intensive. There is an increasing interest in using automated tools which can overcome these limitations and allow accurate and high throughput analysis of animal social behavior.

Several automated analysis tools are based on an estimation of the posture and position of animals. Therefore, the quality of tracking algorithms has a large impact on assessment accuracy (Lorbach et al., 2015). Visual object tracking is a fundamental and crucial problem in machine vision with several real-world applications (Yilmaz et al., 2006; Smeulders et al., 2013). Despite rapid progress in computer vision, tracking animals in their natural environment is still a daunting task due to factors like illumination changes, background clutter, occlusion from vegetation and other natural features, shape deformation, and abrupt motion (Dell et al., 2014). Within controlled laboratory settings, some of those challenges can be alleviated.

Currently, several methods exist for tracking animal locations in laboratory settings, and some have the added benefit of tracking key anatomical features. Approaches that use radio-frequency identification (RFID) (Galsworthy et al., 2005; Lewejohann et al., 2009; Schaefer and Claridge-Chang, 2012) can maintain individual identity well, and thus provide accurate tracking of positions of multiple animals. However, they do not provide information on the positions of important body regions. Techniques combining RFID and video tracking (Weissbrod et al., 2013; de Chaumont et al., 2019) or video tracking visually marked animals (Ohayon et al., 2013; Lorbach et al., 2018) offer both tracking and keypoint locations. However, animals need to go through additional preparations for these methods, adding cost of time and potential confound factors such as discomfort or inflammation. There are also concerns that these methods could alter animal behavior (Burn et al., 2008; Hurst and West, 2010; Walker et al., 2013; Hershey et al., 2018).

Deep-learning-based methods can track animal positions, as well as extract key anatomical points such as the nose and tail-base, which are useful for further behavioral analysis. Inspired by the success of convolutional neural networks (CNNs) in many computer vision tasks (Simonyan and Zisserman, 2014; He et al., 2016; Krizhevsky et al., 2017), several approaches have been proposed to utilize hierarchical features learned from CNNs for visual tracking (Fan et al., 2010; Wang and Yeung, 2013; Danelljan et al., 2015; Ma et al., 2015; Qi et al., 2016; Yang and Chan, 2017). Recently, such techniques have been applied to animal pose estimation (Mathis et al., 2018; Graving et al., 2019; Pereira et al., 2019). Furthermore, the latest version of DeepLabCut (DLC; Lauer et al., 2022) allows tracking features of multiple identical-looking animals, and Social LEAP Estimates Animal Pose (SLEAP) (Pereira et al., 2022) provides a framework for multi-animal body part position estimation. While having stimulated tremendous progress in the field, applying these frameworks on large datasets often requires iterative refinement using a large training set and/or human correction in post-processing. This is due to swapped or missed features and identities caused by visual occlusion during social interactions. While there is a growing number of public repositories and datasets for behavioral video recordings and pre-trained machine learning models for analyzing such recordings (Sun et al., 2021; Open Behavior Video Repository, 2023; Ye et al., 2023), there are limited publicly available labeled datasets with socially interacting mice of identical appearance for developing new techniques and comparing different approaches.

Here, we propose a markerless approach which combines a conventional tracking method and segmentation based on deep learning for tracking the identities of two interacting mice of the same appearance. We focused on controlled experimental settings, so we can start with high-quality video data. Additionally, we used top-down views of mice and considered only simple interactions including different types of sniffing, touching, and locomotion. Within these constraints, we sought to improve tracking accuracy and limit identity switches without increasing human effort in training or post-processing. Specifically, we use Mask R-CNN (He et al., 2017), a flexible and general framework for object instance segmentation, to segment the mice when they interact with each other. To train the model effectively, segmented images of the animals in a wide variety of postures are needed. We took advantage of the high contrast in our chosen imaging settings to automatically create one of two training datasets without any human annotation. A second training set was created using manual segmentation of images when the animals are close together. After segmentation, we used a hand-designed module to track the snout and tail-base of both animals, which are important for quantifying social behaviors such as anogenital, head/face and body sniffing, and touching and trajectory-based parameters such as distance traveled. Finally, we incorporated a deep-learning-based pose estimation approach, such as DLC or SLEAP, to further mitigate issues such as lost key points due to occlusion, which are common when the animals are in close contact.

To validate the proposed system and test the extent of generalization, we trained the algorithm using images from a simple, clutter-free setup. We then applied it to videos collected in different relevant setups with potentially confounding variables such as bedding and fiberoptic or electrophysiology implants. The results were then evaluated using previously established metrics and compared with the state-of-the-art automated methods and human annotation. Results indicated that our proposed approach is effective within our chosen constraints of imaging settings and experimental setups. Specifically, we reduced snout and tail-base location errors and the number of identity switches by 1–2 orders of magnitude and improved multi-object tracking accuracy (MOTA) by ∼15% compared to using DLC or SLEAP alone. Ensembling our algorithm with DLC led to further improvement.

To demonstrate the utility of this approach, we used our system to address a biological question. It is known that mice and rats typically show a preference for social novelty, spending more time investigating an unfamiliar versus a familiar conspecific (Moy et al., 2004; Oettl et al., 2016). To discriminate between familiar and unfamiliar conspecifics, rodents rely on olfactory cues (Sanchez-Andrade and Kendrick, 2009; Oettl et al., 2016). Here, we asked how social discrimination of a familiar versus an unfamiliar conspecific will be affected by loss of olfaction, i.e., anosmia. We used intranasal ZnSO_4_ to induce anosmia in some animals (Ducray et al., 2002), while control animals received intranasal saline. We then assessed how anosmic and control mice behaved toward familiar or unfamiliar conspecifics. From the automated analyses, we were able to make several conclusions regarding how anosmia impacts mouse social behaviors toward familiar and unfamiliar conspecifics.

## Materials and Methods

### Experimental setups and MT dataset

**Figure 1.**
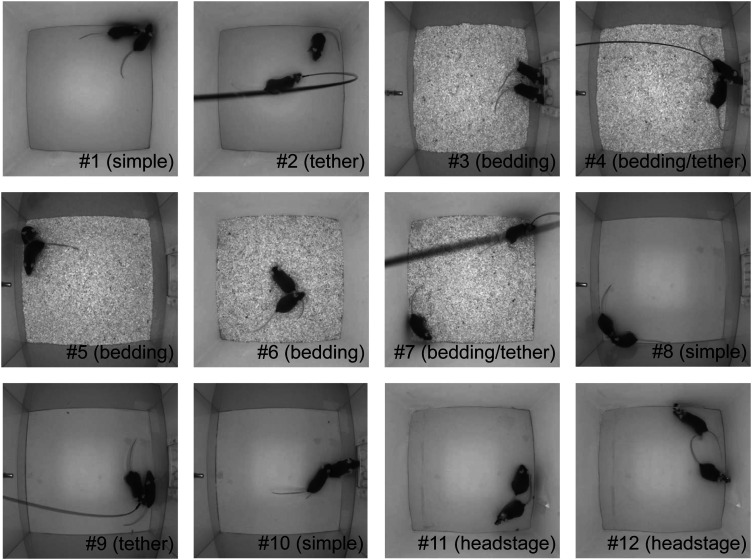
Snapshots taken from the videos illustrating 12 experimental setups used in the mouse tracking (MT) dataset. Details for each of the settings are in Table 1.

Experiments were conducted in 12 different settings as shown in Table 1. Figure 1 shows sample images from the videos highlighting the different settings. In all cases, the animals were placed in a cage measuring 30 × 30 × 30 cm^3^ with the walls lined with a diffuse material. A camera was installed on the lid of the cage and pointed toward the bottom of the cage. Infrared lights on the cage lid and an infrared bandpass filter on the camera were used to ensure constant image brightness regardless of ambient lighting and maintain good contrast. All the videos were recorded at 30 frames per second with a resolution of 540 × 540 pixels. A total of 14 C57BL/6J, BTBR, or Crh-IRES-Cre::Ai14 mice, 9 female and 5 male, aged 7–20 weeks were used for the experiments. Mice were housed in standard mouse cages with access to food and water ad libitum, in a humidity- and temperature-controlled room with a 12-h light/dark cycle. All procedures for this study were performed according to the recommendations by the Canadian Council for Animal Care. The protocol of this study was approved by the Health Sciences Animal Care Committee of the University of Calgary.

**Table 1.** Details of the experimental settings used in the MT dataset

Video	Animal genotype	Sex	Bedding	Food and water	Implant
1	C57BL/6, C57BL/6	F/F	–	–	–/–
2	Crh-IRES-Cre::Ai14, C57BL/6	F/F	–	–	Fiberoptic/–
3	C57BL/6, C57BL/6	F/F	Yes	–	–/–
4	Crh-IRES-Cre::Ai14, C57BL/6	F/F	Yes	Yes	Fiberoptic/–
5	C57BL/6, C57BL/6	M/F	Yes	Yes	–/–
6	BTBR, BTBR	M/F	Yes	–	–/–
7	Crh-IRES-Cre::Ai14, C57BL/6	F/F	Yes	–	Fiberoptic/–
8	C57BL/6, C57BL/6	F/F	–	Yes	–/–
9	Crh-IRES-Cre::Ai14, C57BL/6	F/F	–	Yes	Fiberoptic/–
10	C57BL/6, C57BL/6	M/F	–	Yes	–/–
11	C57BL/6, C57BL/6	M/F	–	–	EEG/–
12	C57BL/6, C57BL/6	M/M	–	–	EEG/EEG

Four animals were implanted with fiberoptic or electroencephalography (EEG) headstages. Twelve videos, each 10 min long, were obtained. We term this group of videos as the MT dataset. For analysis, videos were grouped into four categories based on commonly encountered experimental settings—simple (videos 1, 8, and 10), with bedding (videos 3–7), with tether (videos 2, 4, 7, and 9), and with a headstage (videos 11 and 12).

### Overview of the proposed approach

**Figure 2.**
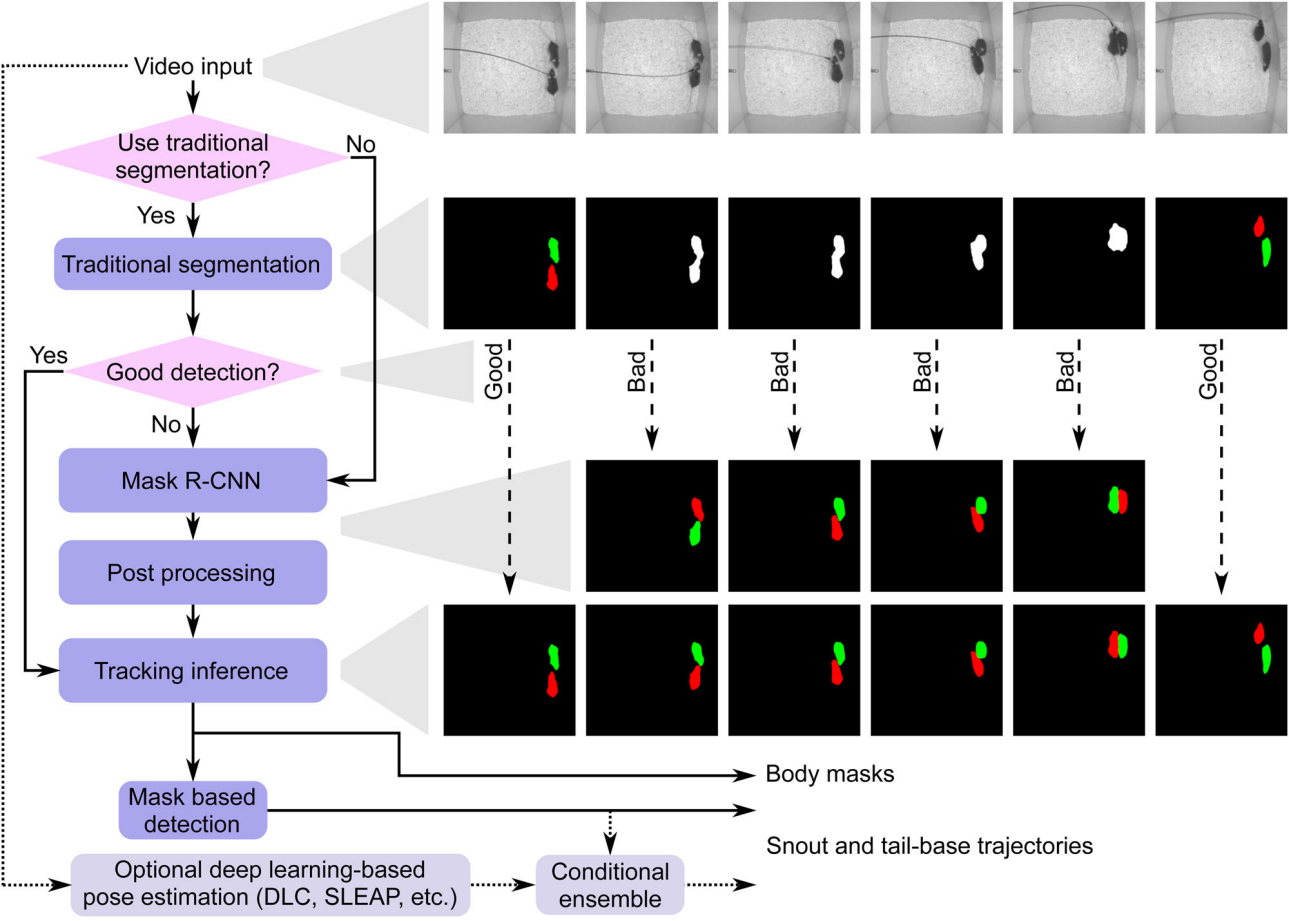
The pipeline of the proposed algorithm for MT and feature detection after training is complete. Traditional segmentation (details in Extended Data Fig. 2-1) can be optionally used to reduce computational cost. User can select to bypass this and use Mask R-CNN exclusively. Our approach generates body masks and snout and tail-base coordinates. It can optionally be ensembled with deep-learning-based pose estimation techniques such as DLC or SLEAP for further improvement of snout and tail-base detection.

Figure 2 shows the top-down approach of our proposed algorithm. Within our constraint of controlled imaging conditions, we can often ensure good contrast. As a result, we can significantly reduce computational cost by optionally using traditional approaches to segmentation. In such cases, input sequence of video frames first go through a stage of conventional foreground detection. In this stage, a background model is created, either using images from before the experiment started or using a temporal median filter on the very first frames after the experiment starts. The foreground is obtained by thresholding with a threshold model which is chosen to be half of background intensity, followed by morphological operations (Extended Data Fig. 2-1). Subsequently, the frames where the number of foregrounds is equal to the number of animals bypass the next step. Tuning thresholding and morphological parameters is incorporated into our implementation of the pipeline. It should be noted that this step is completely optional and can be skipped if imaging conditions are poor or inconsistent, or if the user does not want to tune parameters.

10.1523/ENEURO.0154-22.2023.f2-1 Figure 2-1 Foreground detection includes background subtraction, thresholding and a series of morphological operations: closing (9-pixel-radius circular structuring element) and opening (3-pixel radius circular structuring element). Download Figure 2-1, TIF file.

Even in good imaging conditions, traditional segmentation approaches will fail when the animals are in close physical proximity. These frames, or all the frames if the traditional approach is not used, are sent to the Mask R-CNN model to segment the mice. The areas of output blobs generated by the Mask R-CNN are then verified to filter out segmentation errors in the post-processing stage. In the next stage, tracking inference, the foreground masks of all frames are matched to subsequent frames to obtain the trajectories of each animal.

Finally, the snout and tail-base are detected by a handcrafted approach termed mask-based detection (MD). We also ensemble the outputs of our method with a deep-learning-based pose estimation method. While any such technique can be used, we tried with DLC and SLEAP, and present results with the former due to better tracking accuracy with our data. In the following sections, we elaborate on the details of key modules and evaluation.

### Data preparation

**Figure 3.**
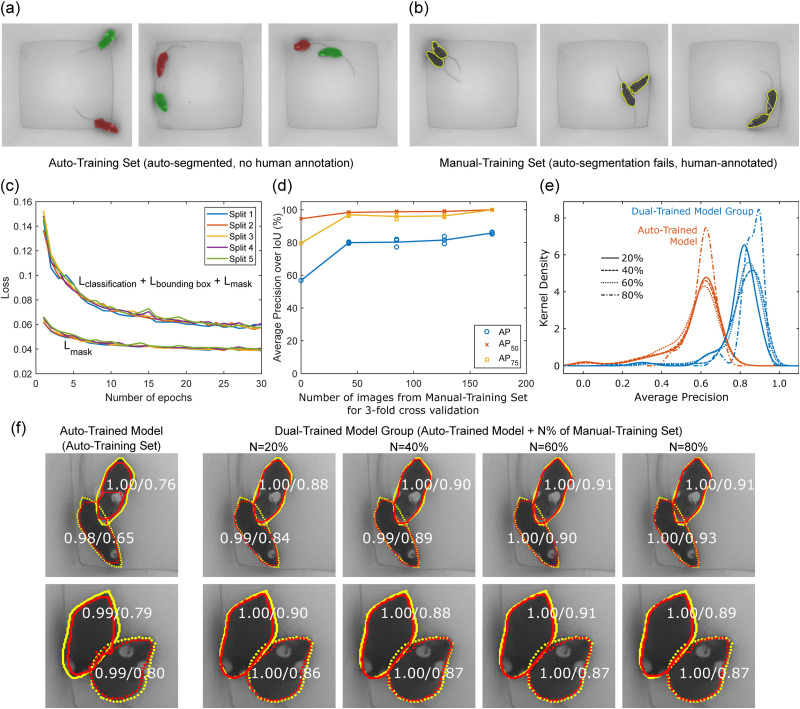
Training and evaluation of the Mask R-CNN. ***a***, Example images with labels taken from the auto-training set. ***b***, Example images with human annotations taken from the manual-training set. Extended Data Figure 3-1 compares human annotation required for all approaches. ***c***, Mask, bounding box, and classification losses for fivefold cross-validation on the auto-training set in which the dataset was split into five sets, the model trained on four splits, and validated on the left-out split. ***d***, Average precision metrics (*AP*, *AP*_75_, and *AP*_50_) of test data for three splits of training data vs. number of training images corresponding to 0, 20, 40, 60, and 80% of the manual-training set. ***e***, Kernel density estimate of *AP* of the auto-trained model and dual-trained model groups on test data for the first split of the training set which account for 20–80% of the manual-training set. ***f***, Visualization of the outputs of the auto-trained model and dual-trained model groups with 20, 40, 60, and 80% training fraction from the manual-training set (red) and human annotation (yellow). Each row shows performance on a different frame. Number pairs are predicted mask confidence and IoU.

Iterative training with data drawn from a variety of expected test scenarios is suggested in all deep-learning-based tools (He et al., 2017; Lauer et al., 2022; Pereira et al., 2022) for improving generalizability and performance. However, we chose to work with a limited set of experimental settings and wanted to improve performance within those constraints with a fixed cost for human annotation. We generated training sets from a separate 7 min video recorded in the most minimalistic setting, i.e., with two C57BL/6 mice with no implants without bedding or any other enrichments. Since there are no confounders in this experimental setting, it achieves the best foreground separation through conventional background subtraction. Two foregrounds were obtained, i.e., both mice were successfully segmented, in about 84% of the frames. We randomly selected 10% of these to create a training set we term the auto-training set (1,060 frames). It should be noted that the auto-training set is obtained with no human annotation. In addition, we randomly chose ∼10% of the video frames (212 frames) where the conventional foreground detection algorithm failed to separate mice due to physical proximity. We then manually segmented those frames using the Labelme annotation tool (Wada, 2023) to build a second training set we term the manual-training set. Several images taken from both these training sets are shown in Figure 3, *a* and *b*. Both training sets were used to train the Mask R-CNN. To train DLC and SLEAP, a second manual-training set was created from seven different 1-min videos also recorded in the most minimalistic setting, but with different lighting and with different pairs of C57BL/6 mice with no implants. Between 30 and 32 images were taken from each video for a total of 212 images including images with closely interacting animals.

10.1523/ENEURO.0154-22.2023.f3-1 Figure 3-1 Example human annotations required for training DLC (top row), SLEAP (middle row), and our approach (bottom row). While annotation styles are different, human effort and time for both is comparable. Download Figure 3-1, TIF file.

Extended Data Figure 3-1 shows example human annotation required for all approaches. For optimal DLC performance, we manually annotated 12 keypoints—snout, left ear, right ear, shoulder, spine 1, spine 2, spine 3, spine 4, tail-base, tail 1, tail 2, and tail end—in each training image (Lauer et al., 2022). For SLEAP, we annotated five points per animal (Pereira et al., 2022). Training for our approach requires marking the outline of the body. It should be noted that while the styles of annotation used to prepare the training data for DLC, SLEAP, and for Mask R-CNN are different, in our experience, both took a similar amount of time and effort.

### Mask R-CNN training

Mask R-CNN is one of the best algorithms for object instance segmentation (He et al., 2017). The results achieved by Mask R-CNN have surpassed single model results on the 2016 common objects in context (COCO) instance segmentation competition (Lin et al., 2014). To obtain this performance, the deep neural network was trained on a huge dataset including a total of 2.5 million labeled instances in 328 thousand images. Thanks to transfer learning, rich features learned on huge datasets such as ImageNet (Deng et al., 2009) can be used in other applications by fine tuning on a new, much smaller, dataset. Using the same spirit of transfer learning, we started from the model trained on COCO by Matterport (Abdulla, 2017) and fine-tuned the network on our datasets. Particularly, we did not train all layers, and adjusted only the mask branch. We first fine-tuned the model on the auto-training set with a learning rate of 0.005 using stochastic gradient descent. Figure 3*c* shows the total loss and mask loss on the validation set during the training process using fivefold cross-validation. The model converges around epoch 25. We term the model trained on the auto-training set, the auto-trained model. We then tested this model on the manual-training set which had the ground truth created by human annotators. We evaluated the model using three standard segmentation metrics *AP*, *AP*_75_, and *AP*_50_ (COCO, 2023), where AP denotes average precision which is defined as the area under the precision recall curve (PR curve). The PR curve is formed by a set of precision (the ratio of true to predicted positives) and recall (the ratio of true to actual positives) pairs. These are obtained by changing the score cutoff from 50 to 95% at a step of 5% for the intersection over union (IoU), a metric that measures the degree of overlap between a blob generated by the model and its ground truth. *AP*_75_ and *AP*_50_ are obtained when the IoU threshold is fixed at 75 and 50%.

The auto-trained model, without training on any images from the manual-training set, i.e., without ever seeing mice in close physical proximity, achieved an *AP*, *AP*_75_, and *AP*_50_ of 58, 80, and 95%, respectively, as shown in Figure 3*d*. Next, we investigated the network when trained on images with animals close to each other from the manual-training set. We varied the size of the training set from 20, 40, 60 to 80% of the full set (3 splits for all training set fractions: 2 for training and 1 for test) and fine-tuned the auto-trained Model 12 times on them. We name these 12 models the dual-trained model group. The performance of the new networks improves when trained on 20% of the manual-training set and then plateaus as seen in Figure 3*d*. Figure 3*e* shows a closer look at performance on one split for different training sizes. The set of blue lines show the kernel density estimate of *AP* achieved by the dual-trained model group on the first splits of training sets which account for 20–80% of the manual-training set. The group of red lines show the performance of the auto-trained model on the same splits as above. Note that for each split, dual-trained model group is obtained by fine tuning the auto-trained model on the other two splits. Improvements from the auto-trained model to dual-trained model group for increasing training from the manual-training set within the dual-trained model group can be seen. Each row of Figure 3*f* visualizes the boundary of the output blobs generated by the models (red) in comparison with human annotations (yellow). The pair of numbers for each animal are the confidence levels of the body mask that the model predicts and the IoU between the mask and its ground truth. A large improvement can be seen between the first and second images which correspond to the auto-trained model and the dual-trained model group trained on 20% of the manual-training set. After that, the performance improves slowly for the dual-trained model groups trained on 40, 60 and 80% of the training set. The results show that only a small number of manually annotated frames in the manual-training set can boost the performance significantly.

### Tracking inference

Once masks of the animals are obtained, individual trajectories were inferred by stitching masks with the most overlapped area together. The process for this is outlined below. Here, $M_{n}^{1}$ and $M_{n}^{2}$ refer to the body masks returned by the algorithm and $ID_{n}^{1}$ and $ID_{n}^{2}$ refer to animal identities that are to be assigned. IoU is the intersection over union and *n* is the frame index.

$$\begin{array}{rl} 1\;:\; & ID_{1}^{1}\leftarrow M_{1}^{1};\;ID_{1}^{2}\leftarrow M_{1}^{2}\\ 2\;:\; & for\;n=2\;to\;N\\ 3\;:\; & \;\delta^{1}=\mathrm{IoU}\;\left(ID_{n-1}^{1},M_{n}^{1}\right)-\mathrm{IoU}\;\left(ID_{n-1}^{1},M_{n}^{2}\right)\\ 4\;:\; & \;\delta^{2}=\mathrm{IoU}\;\left(ID_{n-1}^{2},M_{n}^{1}\right)-\mathrm{IoU}\;\left(ID_{n-1}^{2},M_{n}^{2}\right)\\ 5\;:\; & \;if\;\left|\delta^{1}\right|>\left|\delta^{2}\right|\\ 6\;:\; & \;\;if\;\delta^{1}>0\\ 7\;:\; & \;\;\;ID_{n}^{1}\leftarrow M_{n}^{1};\;ID_{n}^{2}\leftarrow M_{n}^{2}\\ 8\;:\; & \;\;else\\ 9\;:\; & \;\;\;ID_{n}^{1}\leftarrow M_{n}^{2};\;ID_{n}^{2}\leftarrow M_{n}^{1}\\ 10\;:\; & \;\;end\;if\\ 11\;:\; & \;else\\ 12\;:\; & \;\;if\;\delta^{2}>0\\ 13\;:\; & \;\;\;ID_{n}^{1}\leftarrow M_{n}^{2};\;ID_{n}^{2}\leftarrow M_{n}^{1}\\ 14\;:\; & \;\;else\\ 15\;:\; & \;\;\;ID_{n}^{1}\leftarrow M_{n}^{1};\;ID_{n}^{2}\leftarrow M_{n}^{2}\\ 16\;:\; & \;\;end\;if\\ 17\;:\; & \;end\;if\\ 18\;:\; & end\;for \end{array}$$

For frames that had segmentation failures, masks were replaced with the masks of the closest frame that was successfully segmented.

### Detection of snout and tail-base

We further detected the snout and tail-base, which are crucial for recognizing social behaviors such as anogenital investigation and head/torso investigation and quantifying locomotion. Existing approaches mainly rely on detecting users-defined key points to estimate poses before stitching them to form trajectories for each animal (Lauer et al., 2022; Pereira et al., 2022). Such bottom-up methods work well with the visibly articulated animals such as flies and bees (Pereira et al., 2022). However, swapping, lost body parts, and identities switches are common problems when the animals are in close contact or when body parts are occluded . This requires intensive human correction and iterative refining. To cope with these problems, we developed a hybrid method which combines deep-learning-based pose tracking and a mask-based inference. Our approach permits the incorporation of any deep learning pose estimation framework. We used DLC and SLEAP and based on tracking performance, chose DLC. For mask-based inference, we created a handcrafted detection module, termed MD, that can assist DLC in the cases of occlusion and lost key points effectively. Also, the output of the tracking inference plays an important role in maintaining correct identities associated with detected body parts.

#### Mask-based detection

This method is based on observations made in our previous work (Le et al., 2019). Specifically, the algorithm iterates over all frames and estimates the snout and tail-base location by operating on the masks to decide whether the animal’s body is parallel to the floor. This is defined by the length of the animal mask being greater than the median length over all frames and 100% overlap of the body masks with the cage floor. Otherwise, the end points are distinguished by stitching to the closest points on the previous frame.

#### DLC and SLEAP

A DLC model and a SLEAP model were trained only one time using the training sets described in the data preparation section above. We did not use the tracklet refining function for DLC nor the swap correction in either tool at the inference stage. This is because we aim to ensemble the outputs of individual systems to develop a fully automatic tracking tool in which errors would be automatically corrected. The trained DLC and SLEAP models output the same set of points as their respective training data, which are used for pose estimation in those pipelines. Our approach, designed for a smaller subset of behaviors, does not estimate the posture of the animal, and outputs only the body mask, snout and tail-base. To compare keypoint tracking accuracy, we used the snout and tail-base DLC and SLEAP outputs. Other outputs are retained for any user-defined purpose, for example, pose estimation that is not included in our approach.

#### Conditional ensemble

The conditional ensemble relies on the tracked body masks obtained in the tracking inference stage to output the final coordinates of snout and tail-base of each animal. Snout and tail-base coordinates generated by the deep learning framework were chosen if they were both inside an animal body mask. Otherwise, the algorithm selects the MD results.

#### Code accessibility

The 12 videos in the MT dataset and an implementation of our method are available at https://github.com/MaSoMoTr/MaSoMoTr for the research community to further develop tracking and key point detection for automated analysis of social behaviors.

## Results

Here, we validate our approach and compare it to DLC and SLEAP within our constraints of controlled experimental settings and simple social interactions. The human effort in annotating images of closely interacting animals used to train both approaches was similar, and no *post hoc* manual corrections were done for any method. We also consider our methods’s generalizability to more than two mice. Finally, to demonstrate its utility and the kinds of analyses that are enabled by our approach, we quantify certain social behaviors of control or anosmic mice paired with familiar or unfamiliar mice.

### Cross-setup validation

We evaluated the proposed algorithm on the MT dataset, specifically its performance on segmentation, identity tracking, and snout and tail-base detection.

#### Segmentation

**Figure 4.**
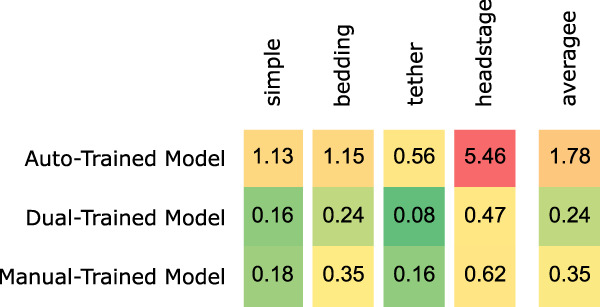
Percentage of frames with segmentation errors over 12 videos in 4 categories in the MT dataset. The auto-trained model is built using the auto-training set that required no human effort and does not have mice in close proximity. The dual-trained model is a fine-tuned version of the auto-trained model incorporating manually segmented images of closely interacting mice from the manual-training set. Manual-trained model is trained only by the manual-training set.

We compared the performance of the algorithm using the auto-trained model and the model obtained by fine tuning it on 80% of the manual-training set, which we term the dual-trained model. The models output two body masks and confidence levels associated with each mask being a mouse. Frames where at least one confidence level was less than 0.9 or where at least one mask area was less than 20% of the largest mask area were considered as failed frames, where the two mice could not be separated. Figure 4 shows the percentage of failed frames over 12 videos in 4 categories in the MT dataset. The algorithm using the dual-trained model failed in less than 1% of the frames across all videos and always outperforms the algorithm using the auto-trained model. This again confirms the previous result that training the Mask R-CNN on frames with both mice in close proximity improves the segmentation performance. Conversely, we also considered whether the auto-training set contributed to the dual-trained model. To answer this, we skipped training on the auto-training set, fitted a model directly on 80% of the manual-training set, and termed the resulting model the manual-trained model. For all the categories in the MT dataset, the performance of the approach with the manual-trained model was not as good as the one with the dual-trained model. Subsequent results are from the dual-trained model.

#### Identity tracking and snout and tail-base detection

To validate the detection of the snout and tail-base, we manually annotated the snout and tail-base of the two mice every 10 frames in all the videos. The annotations were done by two human labelers with the split of 50:50 for each video. The coordinates of snout and tail-base obtained by MD, DLC, SLEAP, and the ensemble were compared with the human generated labels. For the most part, we followed a previously described evaluation protocol (Iqbal et al., 2017) to validate multi-animal pose tracking. To evaluate whether a body part was located correctly, we adopted the widely used PCKh (head-normalized probability of correct keypoint) metric (Zhang et al., 2019), which considers a key point to be detected correctly if it is within a certain threshold from the human annotated location. Since our videos were recorded with a fixed field of view, we apply a fixed threshold for all the animal across videos. Accordingly, we measured the length of the head as the distance from the snout to the midpoint of the shoulders in 212 randomly selected images from Dataset 2 and set the threshold to be 50% of the average head length. In addition, we evaluated center points, defined as the midpoint between the snout and tail-base, which can represent animal identity. We considered the snout, the tail-base, and the center point as individual targets and computed the MOTA (Bernardin and Stiefelhagen, 2008) which is widely used for evaluating multi-target tracking (Milan et al., 2016; Iqbal et al., 2017). This metric is derived from three types of error counts: missed targets (*m*_*n*_), the number of instances where a target is not located or is too far from the actual location; false positives (*fp*_*n*_), the number of instances where a target is located, but more than a threshold distance away; and mismatches (*mme*_*n*_), the number of instances where targets are identified but assigned incorrect identities.

(1) $$\mathrm{MOTA}=1-\frac{\sum_{n}m_{n}+fp_{n}+mme_{n}}{\sum_{n}g_{n}},$$

*g* is the number of targets and the subscript *n* indicates the frame number.

**Figure 5.**
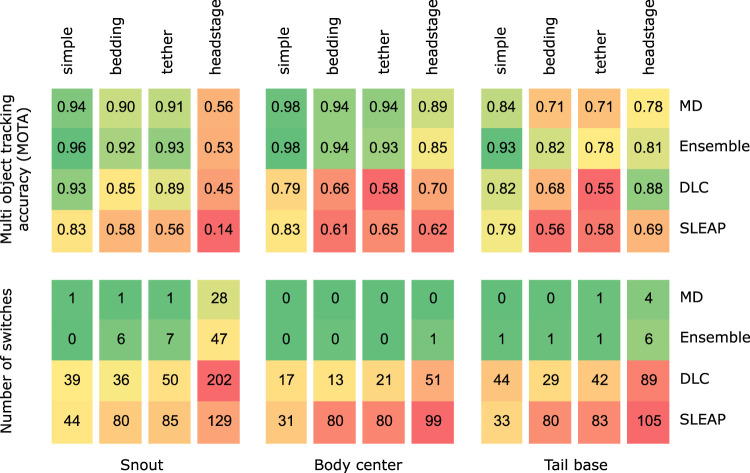
Comparison of our approaches—MD and ensemble—with DLC and SLEAP. The upper panel shows average MOTA and the lower panel shows total instances of switched identities across all 12 videos in the 4 categories.

The upper panel of Figure 5 shows MOTA calculated for the snout, tail-base, and center using the py-motmetrics framework (py-motmetrics, 2023). The number of identity switches that occurred in each video after analyzing with the different methods is shown in the bottom panel. On average, MOTA increases by ∼15% over DLC or SLEAP using our approach. Instances of switched identities are reduced by over an order of magnitude. Since DLC showed higher MOTA than SLEAP, we ensembled our results with DLC and this further improves MOTA. The worst performance, both in terms of MOTA and the number of switched identities, is seen in the headstage group of videos, particularly for the snout due to frequent occlusions caused by the headstage.

**Figure 6.**
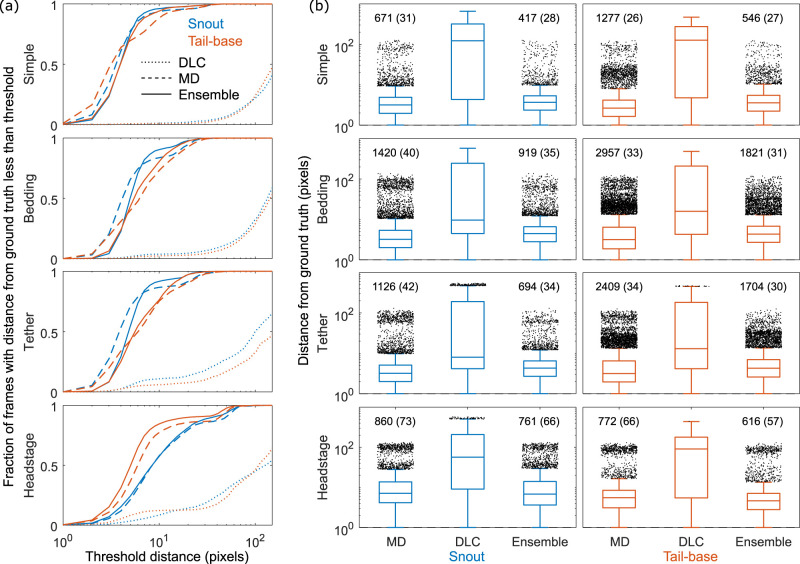
Performance across 12 videos in 4 categories. ***a***, Fraction of frames with the mean distance between model predictions and human annotations below a varying threshold. ***b***, Boxplots showing errors in MD, DLC, and ensemble models. Plots show median, 25th and 75th percentile, and outliers defined as >75th percentile +1.5 times the inter-quartile range. Text above outliers show number of outliers and average outlier within parentheses. Results for individual videos are shown in Extended Data Figures 6-1–6-3.

Figure 6 illustrates average algorithm performance on the four groups of videos in the MT dataset, specifically regarding error distances from the ground truth. The left panel shows the fraction of frames within a certain error distance threshold. Our approach outperforms DLC in all cases. Ensembling worsens the performance somewhat in all but the headstage group, however its benefits can be seen in the right panel which shows a distribution of errors across all frames. While the median errors are a bit higher, all groups show the effectiveness of the conditional ensemble in reducing the number and size of outliers, thereby improving the overall performance. As seen earlier, the worst performance is in the headstage group especially in the detection of the snout. Results for individual videos are shown in Extended Data Figures 6-1–6-3. Extended Data Movies 1–3 show the segmentation and identity tracking for SLEAP, DLC, and Mask R-CNN for three settings: simple, with a fiberoptic implant, and with bedding and a fiberoptic implant. A reduction in the instances of switched identities can be seen when the animals are closely interacting.

10.1523/ENEURO.0154-22.2023.f6-1 Figure 6-1 Performance across videos 1-4. (a) Fraction of frames with the mean distance between model predictions and human annotations below a varying threshold. (b) Boxplots showing errors in MD, DLC and Ensemble models. Plots show median, 25th and 75th percentile and outliers defined as > 75th percentile + 1.5 times the inter-quartile range. Text above outliers show number of outliers and average outlier within parenthesis. Download Figure 6-1, TIF file.

10.1523/ENEURO.0154-22.2023.f6-2 Figure 6-2 Performance across videos 5-8. (a) Fraction of frames with the mean distance between model predictions and human annotations below a varying threshold. (b) Boxplots showing errors in MD, DLC and Ensemble models. Plots show median, 25th and 75th percentile and outliers defined as > 75th percentile + 1.5 times the inter-quartile range. Text above outliers show number of outliers and average outlier within parenthesis. Download Figure 6-2, TIF file.

10.1523/ENEURO.0154-22.2023.f6-3 Figure 6-3 Performance across videos 9-12. (a) Fraction of frames with the mean distance between model predictions and human annotations below a varying threshold. (b) Boxplots showing errors in MD, DLC and Ensemble models. Plots show median, 25th and 75th percentile and outliers defined as > 75th percentile + 1.5 times the inter-quartile range. Text above outliers show number of outliers and average outlier within parenthesis. Download Figure 6-3, TIF file.

10.1523/ENEURO.0154-22.2023.video.1 Movie 1 Output of SLEAP, multi-animal DLC and our approach (Mask R-CNN) on a simple uncluttered video (video #1 from MT dataset). Download Movie 1, MP4 file.

10.1523/ENEURO.0154-22.2023.video.2 Movie 2 Output of SLEAP, multi-animal DLC and our approach (Mask R-CNN) on a video with one animal with a fiberoptic tether (video #2 from MT dataset). Download Movie 2, MP4 file.

10.1523/ENEURO.0154-22.2023.video.3 Movie 3 Output of SLEAP, multi-animal DLC and our approach (Mask R-CNN) on a video with one animal with a fiberoptic tether and bedding in the arena (video #7 from MT dataset). Download Movie 3, MP4 file.

### Generalization

**Figure 7.**
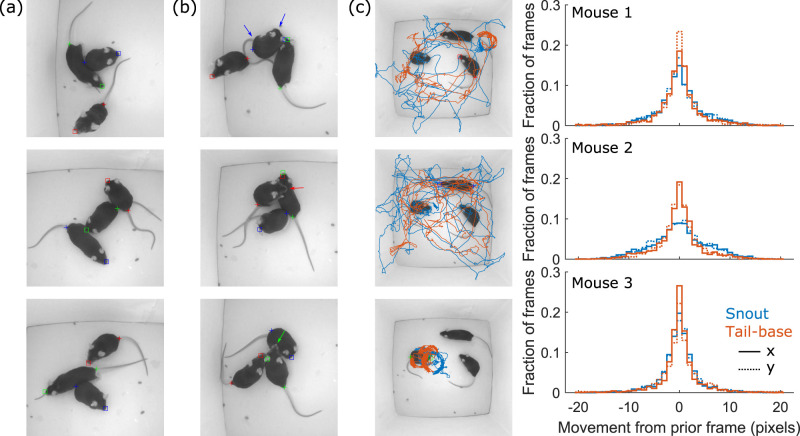
Examples of ***a***, good and ***b***, visibly inaccurate snout and tail-base detection in a group of three mice. ***c***, Snout and tail-base trajectories for each animal along with a distribution of frame-to-frame movement in *x* and *y* coordinates.

We expanded the cross-setup validation to include an experiment with more than two mice. We recorded a video with three BTBR mice interacting for 1  min (1,800 frames). Figure 7*a* shows some examples of good performance of the approach when coping with occlusions, while some errors can be seen in the frames shown in Figure 7*b*. Figure 7*c* shows the trajectories of the snout and the tail-base of the mice detected by the algorithm using the dual-trained model trained as described above. The figure also shows the distribution of the frame-to-frame movements in *x* and *y* coordinates. The absence of large movements suggests continuity in trajectories and accurate tracking.

### Application

**Figure 8.**
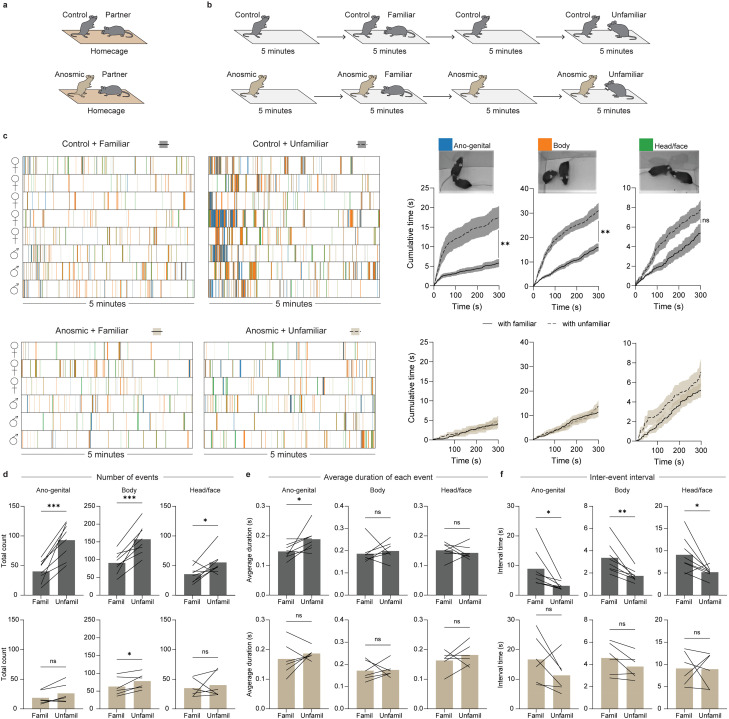
Application of the markerless MT approach showing that loss of olfaction through ablation of the main olfactory epithelium impairs social recognition behaviors in mice. ***a***, Mice were housed in same sex pairs; one mouse per pair was a control (intranasal saline-treated) or anosmic (intranasal ZnSO_4_-treated). ***b***, Experimental paradigm for social recognition test: anosmic/control mice acted as “observers” (Obs) and were presented with either a familiar demonstrator (Dem) or an unfamiliar demonstrator. ***c***, Left: behavioral ethograms of control (top) and anosmic (bottom) observers while interacting with a familiar or unfamiliar demonstrator. Each color indicates when the snout of the observer was directed toward the anogenital (blue), body (orange), or head/face (green) region of the demonstrator. Right: cumulative distributions of each of the social investigative behaviors for control (top) and anosmic (bottom) observers toward familiar (solid line) or unfamiliar (dotted line) demonstrators. ***d***, Total number of social investigation events by control (top) and anosmic (bottom) observers directed toward the anogenital, body, or head/face region of familiar versus unfamiliar demonstrators. ***e***, Average duration of each social investigation event. ***f***, Interval between social investigation events. *p < 0.05; **p < 0.01; ***p < 0.001. Paired *t*-test comparing social investigation toward a familiar versus unfamiliar demonstrator. Analysis of demonstrator mice behavior in Extended Data Figure 8-1.

To test the application of our method, we used it to address a biological question: how does loss of olfaction, through ablation of the main olfactory epithelium, impact social recognition behaviors of mice. A total of 16 female and 12 male Crh-IRES-Cre mice were housed in same sex pairs. One mouse from each pair received 10 μL saline (control) or 1% ZnSO_4_ (anosmic model) into each nostril to ablate the main olfactory epithelium (Ducray et al., 2002), while the partner was untreated (Fig. 8*a*). The next day, control or anosmic experimental mice and their partners were habituated to the tracking arena. The experimental mouse was placed into the arena first for 5 min before the partner was added for an additional 5 min. The following day (48 h post ZnSO_4_ or saline treatment), control or anosmic mice were returned to the tracking arena for a social recognition assay (Fig. 8*b*). The control or anosmic mouse acted as the “observer” (obs) and was placed into the arena first. After 5 min alone, the familiar partner acting as a familiar “demonstrator” (dem) was placed in the arena. After 5 min, the familiar demonstrator was removed. The observer was alone for another 5 min before an unfamiliar demonstrator (same-sex, adult) was placed in the arena for 5 min. The arena was cleaned with 70% ethanol between testing observers.

10.1523/ENEURO.0154-22.2023.f8-1 Figure 8-1 Social investigation behaviors of familiar or unfamiliar demonstrators towards control or anosmic observers. Social investigation behaviors include when the snout of the demonstrator was directed towards the ano-genital, body, or head/face region of the observer. Cumulative distributions show that familiar (solid line) and unfamiliar (dotted line) demonstrators spend similar amount of time engaged in each social investigation behavior when with a control (top) or anosmic (bottom) observer. Download Figure 8-1, TIF file.

The coordinates of each mouse’s snout and tail-base, as well as the body mask, were used to determine social investigation of each mouse toward the other. If one animal’s snout was within a 25-pixel threshold distance of the other animal’s snout, tail-base, or body mask, we assigned a label of head-directed, anogenital-directed, or body-directed sniffing, respectively, to that frame. In order to benchmark this approach, a separate 5-min video was recorded and labeled independently by two human annotators with the three sniffing behaviors: head-directed, body-directed, and anogenital-directed. Annotator 1 was more experienced and considered as the ground truth. Table 2 shows the metrics measuring the overlap between annotators 1 and 2 and the thresholding method with annotator 1.

**Table 2.** Benchmarks for quantifying sniffing behaviors using a distance thresholding approach with snout, tail-base, and body mask coordinates

	Annotator 1 versus annotator 2			Annotator 1 versus thresholding		
	Precision	Recall	F1 score	Precision	Recall	F1 score
Head-directed	0.75	0.53	0.62	0.54	0.54	0.54
Anogenital-directed	0.72	1.00	0.84	0.86	0.68	0.76
Body-directed	0.82	0.86	0.84	0.74	0.88	0.80

Although the automated results averaged across the three sniffing behaviors are 0.06 F1-score lower than the human baseline, they are competitive with previous methods (Lorbach et al., 2015, 2018) indicating that the distance thresholding approach can be applied to quantify simple sniffing behaviors with just the snout, tail-base, and body mask coordinates.

For the olfaction study, the time the snout of each mouse was directed toward the anogenital, body, or head/face region of the other mouse was quantified (Fig. 8 and Extended Data Fig. 8-1). Ethograms of these three different types of snout-directed social behaviors reveal that although saline-treated observers investigate both familiar and unfamiliar demonstrators, there is significantly more investigation if the demonstrator is unfamiliar (Fig. 8*c*). In both situations, the majority of social investigation occurs during the first minute (Fig. 8*c*). In contrast, anosmic observers spend less time investigating their partners and do not show any preference for the unfamiliar demonstrator compared to the familiar demonstrator (Fig. 8*c*). Cumulative distribution graphs of time spent in each of these behaviors confirm that snout-to-anogenital and snout-to-body investigation is increased in control observers when the demonstrator is unfamiliar (Fig. 8*c*). This is not true for anosmic observers (Fig. 8*c*). From the tracked data, we can also determine the number of social investigation events (Fig. 8*d*), the average duration of each event (Fig. 8*e*), and the average time between events (Fig. 8*f*). In control observers, the number of social investigation events increased (Fig. 8*d*) and the inter-event interval decreased (Fig. 8*f*), when the demonstrator was unfamiliar, for all types of investigation (anogenital, body, and head/face). The average duration of only anogenital investigation was increased when the demonstrator was unfamiliar (Fig. 8*e*). For anosmic observers, the number of body-directed investigation events increased when the demonstrator was unfamiliar (Fig. 8*d*), while no other parameters were significantly changed. These findings suggest that intact olfaction is required for many social behaviors that demonstrate social discrimination of a familiar versus unfamiliar conspecific.

Since both animals are tracked, we can analyze demonstrator mouse behavior as well. Demonstrator mice show very little snout-directed social investigation of observer mice. Time spent engaged in these different types of investigation are also not altered if the demonstrator is familiar or unfamiliar to the observer (Extended Data Fig. 8-1). Thus, social investigation behaviors appear to be uni-directional in this case, exhibited predominantly by the mouse that was in the arena first, i.e., the observer.

**Figure 9.**
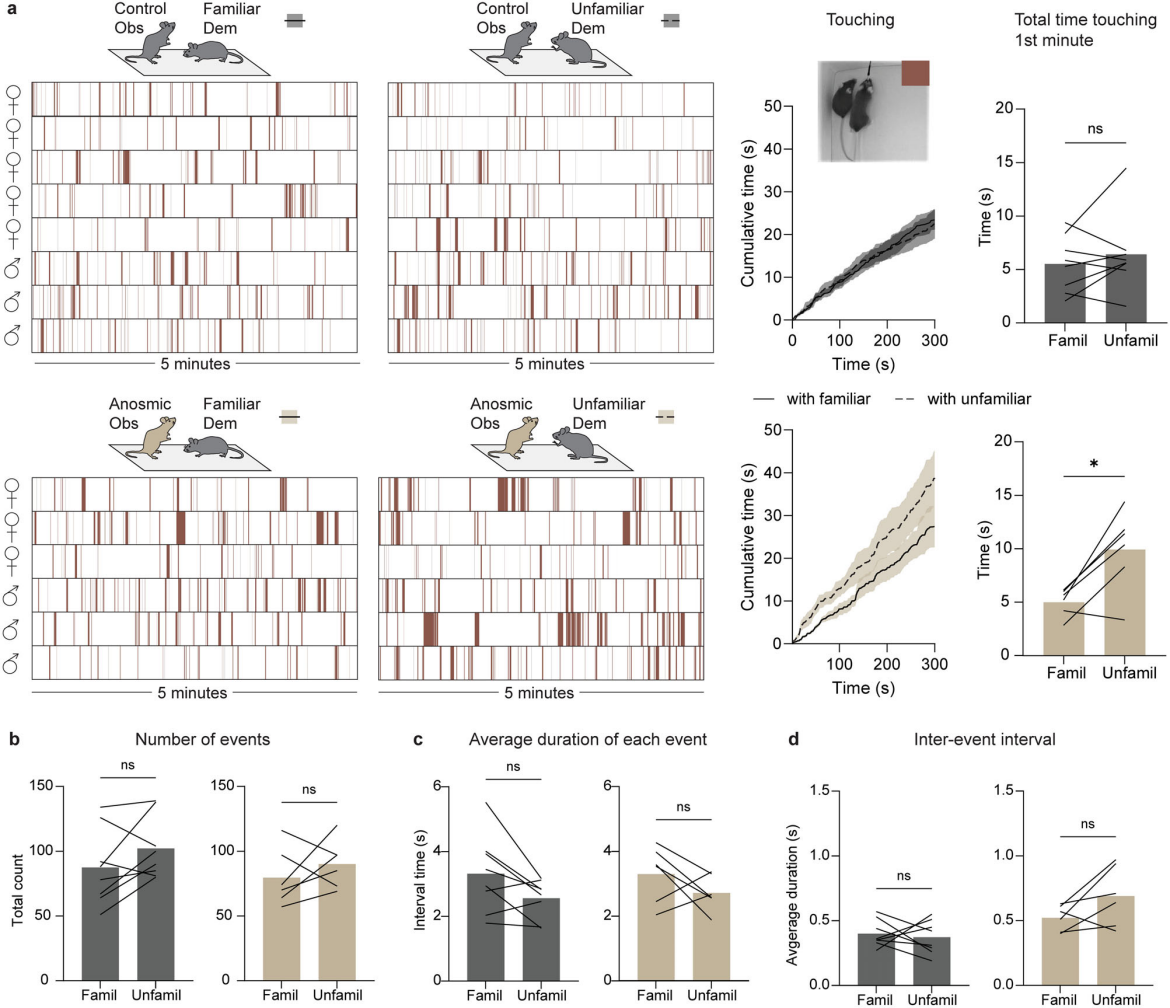
Application of the markerless MT approach showing that anosmic mice spend more time in contact (non-snout-directed contact) with unfamiliar versus familiar demonstrators. ***a***, Left: behavioral ethograms of control (top) and anosmic (bottom) observers while interacting with a familiar or unfamiliar demonstrator. Each brown bar indicates when the mice were in contact (touching) that was not as a result of snout-directed investigation by the observer or demonstrator. Right: cumulative distributions of touching in pairs of control (top) or anosmic (bottom) observers with familiar (solid line) or unfamiliar (dotted line) demonstrators. Bar graph shows total time spent touching during the first minute of the 5-min period. ***b***, Total number of touching events between control (left) or anosmic (right) observers, with familiar or unfamiliar demonstrators. ***c***, The average duration of each touching event. ***d***, The inter-event interval between touching events. *p < 0.05. Paired *t*-test comparing touching between observers and familiar versus unfamiliar demonstrators.

Since our approach generates body masks in addition to the snout and tail-base coordinates, we could also determine how much time observers and demonstrators spent in contact with one another by detecting at what times the shortest distance between the masks was less than a threshold. By removing the snout-directed social investigation times from these data, we could determine the amount of time each pair spent touching that was not because of observer-to-demonstrator or demonstrator-to-observer snout-directed investigation. Behavioral ethograms indicate that pairs of mice in which one is anosmic spend more time in contact compared to control pairs (Fig. 9*a*).

Plotting these data as cumulative distributions shows that anosmic observers spend more time in contact with unfamiliar demonstrators than familiar demonstrators, but only near the beginning of the 5-min period (Fig. 9*a*). Taking the average time spent touching during the first minute shows that anosmic observers do spend more time in contact with unfamiliar demonstrators than familiar demonstrators (Fig. 9*a*). The number of contact events (Fig. 9*b*), average duration of events (Fig. 9*c*), and inter-event intervals (Fig. 9*d*) for the total 5-min period were not altered by the familiarity of the demonstrator. These findings show that anosmic mice might behaviorally discriminate between unfamiliar and familiar conspecifics through changes in bodily contact rather than snout-directed investigation.

**Figure 10.**
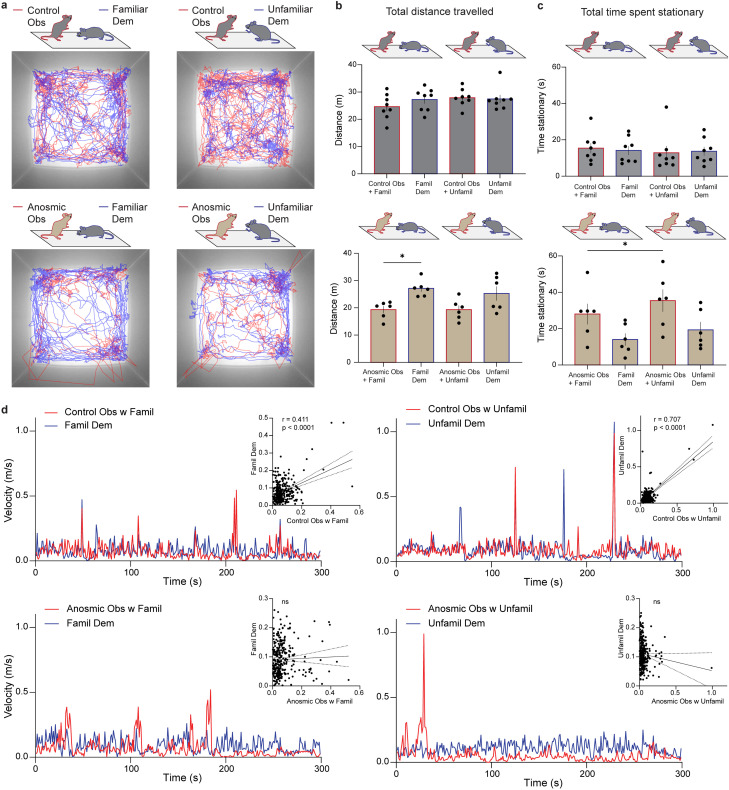
Application of the markerless MT approach showing distance traveled and velocities of pairs of mice (observer + demonstrator). ***a***, An example of raw tracking data of observers (red) and demonstrators (blue), where observers were control (top) or anosmic (bottom) with familiar (left) or unfamiliar demonstrators (right). ***b***, Total distance traveled by observers and their familiar or unfamiliar demonstrators where the observer was control (top) or anosmic (bottom). ***c***, Total time spent stationary by observers and their familiar or unfamiliar demonstrators where the observer was control (top) or anosmic (bottom). ***d***, Examples of velocities (1 s bins) of observers (red) and demonstrators (blue) with familiar or unfamiliar demonstrators where the observer was control (top) or anosmic (bottom). Inset graphs show Pearson correlation analyses of observer velocities versus demonstrator velocities. Data for all animals are shown in Extended Data Table 10-1. *b* and *c*, *p < 0.05, one-way ANOVA with Sidak’s multiple comparisons test.

Tracking the center point of observers and demonstrators shows that when the observer is anosmic, the pair travel a shorter distance than control pairs (Fig. 10*a*). Quantifying the distance covered shows that anosmic observers move less than their non-anosmic demonstrators (Fig. 10*b*), suggesting that loss of olfaction impacts exploratory behaviors in general. Anosmic observers also spend more time stationary when they are in the presence of an unfamiliar demonstrator versus a familiar demonstrator (Fig. 10*c*). This again shows behavioral discrimination of an unfamiliar versus familiar conspecific by anosmic mice that is independent of snout-directed behaviors. Looking at velocity of observer and demonstrator mice in 1s bins over the 5-min period shows that the velocity of control observers and their demonstrators tend to be correlated (75% of pairs correlated, Extended Data Table 10-1; example shown in Fig. 10*d*). Velocity of pairs where the observer is anosmic tend to not be correlated (75% of pairs not correlated, Extended Data Table 10-1; example shown in Fig. 10*d*). This suggests that intact olfaction is required for coordinated behaviors in a pair of mice.

10.1523/ENEURO.0154-22.2023.t10-1 Table 10-1 Velocities (calculated in 1 second bins as metres per second) correlated more frequently in control observer + demonstrator pairs (75%) than in anosmic observer + demonstrator pairs (25%). Download Table 10-1, DOCX file.

## Discussion

Reliably tracking multiple animals is critical for automated behavioral assays evaluating social interactions. However, it is extremely time-consuming to evaluate the quality of animal tracking systematically, because the ground truth needs to be generated manually. As of now, there are no publicly available benchmark datasets, and only a few attempts have been made to evaluate tracking methods (Lorbach et al., 2015; Salem et al., 2015). Some reports have indicated that behavioral assays in close-to-natural arenas are more ethologically relevant than those in bare cages (Castelhano-Carlos et al., 2014; Puścian et al., 2016). Additionally, advances in optogenetics, fiber photometry, and tethered or wireless electrophysiology permit simultaneous physiological manipulation and recording along with behavioral tests. These require animal-mounted headstages and fiberoptic or electrical cables. Thus, tracking algorithms that are robust to the presence of bedding and other enrichments as well as various implants would be particularly useful.

Here, we developed an approach for top-down videos of mice in controlled illumination that combines conventional tracking and a deep-learning-based instance segmentation method. It advances tracking for certain social behaviors, specifically sniffing, touching, and locomotion, in two important ways. First, we decreased the computational cost of deep neural networks that are sufficiently powerful to cope with challenges like social contact between animals to extract animals’ masks but must operate on a frame by frame basis. We took advantage of good contrast and used conventional methods to obtain blobs when the animals were apart and only applied the Mask R-CNN in cases when the animals were interacting. This also reduced the required training set size for the Mask R-CNN since the mouse body is highly deformable and can result in a great number of postures. Second, the conventional method can be implemented in parallel for a batch of frames using multiple CPU cores. Consequently, the hybrid approach is much more time efficient compared to the approach using only Mask R-CNN. However, the hybrid approach does rely on good contrast in the videos. In case of poor or inconsistent illumination, the hybrid approach can be disabled and every image can be segmented using the Mask R-CNN. This was the case in some videos from our anosmia study with less uniform and overall lower illumination. Extended Data Movie 4 shows successful tracking results without the hybrid approach. In addition, the ensemble results of snout and tail-base detection have shown that combining handcrafted inference with the deep neural network can improve the performance of the system considerably, and that the method remains robust with bedding and implants. This suggests that injecting human knowledge can be very helpful to mitigate the limitations of end-to-end approaches.

10.1523/ENEURO.0154-22.2023.video.4 Movie 4 Tracking, snout and tailbase locations returned by our approach for a video in our anosmia study with poorer illumination and contrast compared to MT dataset videos. Download Movie 4, MP4 file.

Current approaches directed to quantifying social behavior have unique advantages and limitations. Researchers need to carefully consider these factors to determine the suitable methods for the unique nature of any experiment. If a reasonable contrast between the background and the animals is maintained, the background appearance can be modeled at every pixel location, and then the foreground can be segmented (Noldus et al., 2002; Branson et al., 2009; Swierczek et al., 2011). This approach, however, is largely limited to individual animals since tracking multiple animals in the same arena is prone to occlusion or “identity switching” because of frequent social interactions at close range.

As mentioned earlier, approaches that use RFID can provide accurate location tracking of multiple animals but do not provide information on the positions of important body regions. RFID methods, requiring surgically implanted RFID tags in the animals, have been used to track individuals in group cages (Galsworthy et al., 2005; Schaefer and Claridge-Chang, 2012; Weissbrod et al., 2013; de Chaumont et al., 2019). This allows for experiments in semi-natural and less controlled environments (Weissbrod et al., 2013) with accessories such as feeders, bridges, and shelters. Besides requiring surgery, another limitation is that integration of detection coils might complicate the testing arena (Lewejohann et al., 2009). Animals also need to recover for a few days post-surgery, which imposes constraints on experimental designs and timelines.

Other approaches have used physical marking of individual animals to assist automatic video-based tracking systems. Human hair bleach has been used to create unique patterns on mouse fur, enabling multi-day tracking (Ohayon et al., 2013). Noldus EthoVision XT 12 (Lorbach et al., 2018), a commercial system, tracks white rats and their body parts after marking animals with a black marker. Although such marking approaches can make tracking algorithms more robust and avoid switching identities, they have drawbacks (Walker et al., 2013). Markings may fade over time requiring reapplication under restraint and handling by humans which is time-consuming and stressful for the animals (Burn et al., 2008; Hurst and West, 2010). Certain compounds in marking agents may also alter behavior in some assays (Burn et al., 2008; Hershey et al., 2018). Furthermore, marking procedures must be determined in advance, and implemented carefully and consistently.

Several markerless tracking methods have been proposed to track animals. One example is carrying out the experiments with a pair of black and white mice (Burgos-Artizzu et al., 2012; Hong et al., 2015). However, this approach is not very broadly applicable since commonly used genetically modified animals are often dark colored (The Jackson Laboratory, 2023), and littermate controls are often of the same fur color as the experimental group.

When multiple animals are involved, social contacts routinely lead to occlusions, causing identity errors. To cope with occlusion, Matsumoto et al. (2013) proposed utilizing multiple 3D depth cameras with different viewpoints to reconstruct 3D positions of rats’ body parts in order to analyze social and sexual interactions. While a depth camera provides additional information about posture and body shape compared to a standard camera, the technique requires a relatively complex setup and the process is computationally intensive. Unger et al. (2017) introduced an unsupervised-learning method to track the animals in an uncluttered setting by learning the shape information of each animal, and then segmenting the animal by matching the current shape with corresponding catalog. The method needs considerable preprocessing including human annotation as the shape catalog has to be built for each animal used in an experiment, and cannot be reused for any other animal.

CNN-based trackers have achieved state-of-the-art performance on benchmark datasets (Smeulders et al., 2013; Wu et al., 2015) and have been applied to pose estimation of mice housed in laboratory cages (Mathis et al., 2018; Graving et al., 2019; Pereira et al., 2019). DLC version 2.2 (Lauer et al., 2022) and SLEAP (Pereira et al., 2022) enable tracking unmarked animals. An important feature of such end-to-end deep learning models is their flexibility: with enough carefully prepared training data, virtually any experimental setting can be handled with asymptotically improving performance. For example, they can be trained to track several species and applied to track user-defined points like whiskers and paws. A major limitation of these models is that they require significant human intervention to correct mistakes such as swapped features when tracking multiple animals with limited training data, as indicated by our test on tracking two interacting mice. Additionally, performance was further reduced on videos in enriched arenas or when animals had implants. With comprehensive training data collection and curation, performance can be improved, but at the cost of significant human effort.

Our algorithm was trained using a single video in a simple, uncluttered setting. We used 1,060 non-curated images, i.e., selected without any human input, with animals apart and 212 automatically selected images when the animals were closely interacting, that were manually segmented. A total of 212 images in the same environment, but from 7 different videos with varied mice and lighting, were used to train a DLC model and a SLEAP model. Images were annotated differently but required similar human effort. Though our approach only tracks the snout and the tail-base, we used all 12 annotations points to train DLC because many features resulting from that have been shown to have significant discriminative power (Lauer et al., 2022). Furthermore, annotating fewer points, but in more images, is unlikely to be beneficial because DLC performance has minimal improvement beyond training with around 200 images (Mathis et al., 2018). We compared the performance in videos with varied experimental settings that included bedding, and animals with fiberoptic or headstage implants. Our results, obtained without any human correction after automated analysis, demonstrated a near elimination of switched identities and a ∼15% improvement in tracking accuracy. Additionally, the results for three mice show the potential to apply our approach to experiments involving a group of mice. Deep-learning-based tools often suggest training with a wide variety of videos covering the range of experimental settings that the tool is expected to encounter. This leads to excellent generalization at the cost of increased human effort in preparing the training data. Our motivation was to limit use cases to commonly encountered settings in experiments involving mice and improve performance with minimal human effort in training. Thus, we trained the Mask R-CNN on frames from only one video in the simplest experimental settings. DLC and SLEAP were trained on similar images, but from seven different and varied videos as instructed in the respective documentation.

By applying our markerless MT tool to address a biological question, we made several discoveries. We specifically tracked the coordinates of two mice, where one mouse was either a control or had been made anosmic. Our results indicated that while control mice show an increase in snout-directed social investigation of a conspecific when the conspecific is unfamiliar versus familiar, this is not true for anosmic mice. Given that rodents rely heavily on olfactory cues in social recognition (Sanchez-Andrade and Kendrick, 2009; Oettl et al., 2016), this result was expected. We found evidence, however, of behavioral discrimination of familiar versus unfamiliar conspecifics in pairs of mice where one was anosmic. Specifically, touching/contact that was not snout-directed investigation from either mouse was increased when the conspecific was unfamiliar versus familiar. We also showed that anosmic mice spend more time stationary when in the presence of an unfamiliar versus a familiar mouse. These subtle differences suggest that anosmic mice use non-olfactory cues to discriminate familiar and unfamiliar conspecifics. Finally, we demonstrate additional value of this tool in tracking velocities of interacting animals. We show that majority of pairs of mice display correlated velocities, indicating coordinated movements; when one mouse is anosmic, however, the pair are less likely to have correlated velocities. Such application of this tool opens doors to many new possibilities of analyses that may hold specific value depending on the research question. Acquiring data on social contact (snout-directed or other), geographic location, and distance traveled, all with temporal resolution, allows for combining of data sets to address questions like: at what velocity does snout-directed social investigation occur? Where in the arena do mice engage in social contact? Do snout-directed social investigations follow a specific pattern? Do mice show reciprocal investigation patterns after a certain time? And how do these all change in mice with a specific genetic mutation, or after an environmental perturbation? While these questions are beyond the scope of the current study, they can now readily be addressed using this tool.

### Limitations

Our method was designed specifically for accurately tracking socially interacting mice, in top-down view videos taken under well-controlled illumination in a purpose-built arena. The algorithm returns the body mask, and locations of only two keypoints, nose and tail-base, for both animals. From these, we can quantify head-nose, body-nose, anogenital-nose and body-body distances, and animal velocities. From this, certain behaviors, such as head-directed, body-directed, and anogenital-directed sniffing, touching, and approach/withdrawal, can be quantified. With these contraints, we tried to minimize training and human *post hoc* correction. As a result, our method is not as flexible as end-to-end deep learning frameworks which, for example, may be more robust to imaging conditions, can be applied to other species, and can estimate animal posture and track arbitrary user-specified body parts.

We focused on simple, non-antagonistic social interactions and have not applied our approach to videos with behaviors such as fighting or mating. In the future, we plan to use outputs from the tool developed in the current work to further classify different social behaviors. It is expected that behaviors such as fighting that involve significant occlusion will be more challenging to classify than sniffing-based behaviors. However, we are optimistic about our approach, because the videos we have analyzed in the current work do include close interactions of two mice such as one crawling over or under the other and our approach did well in following the identities of the two mice.

One limitation in the MD-based location of snouts and tail-bases is our estimation of body length based on the median mask length over all frames in a video. If animals spend a majority of the time in activities such as rearing or being curled-up, the median mask length will be a poor estimate of body length. In our videos, animal behavior was sufficiently variable, but this may be a problem in certain experimental situations. A more specific limitation of our method is that extensive occlusion caused by big implants such as EEG headstages is still a challenge. Thus, in challenging situations, manual validation for identity assignments in tracking results is recommended.

### Conclusion

We proposed a hybrid approach for tracking mice and their snouts and tail-bases in experiments involving two mice of the same color interacting under controlled illumination in a purpose-build arena. We evaluated the method using cross-setup videos in two aspects: tracking identities and snout and tail-base detection. The results obtained with a small number of training images and without any human correction have shown the utility of the proposed method.

## Synthesis

Reviewing Editor: Mark Laubach, American University Decisions are customarily a result of the Reviewing Editor and the peer reviewers coming together and discussing their recommendations until a consensus is reached. When revisions are invited, a fact-based synthesis statement explaining their decision and outlining what is needed to prepare a revision will be listed below. The following reviewer(s) agreed to reveal their identity: NONE. Note: If this manuscript was transferred from JNeurosci and a decision was made to accept the manuscript without peer review, a brief statement to this effect will instead be what is listed below.

All revisions were satisfactory and the paper was judged to have been mush improved by the review process.
