# Supplementary figures and images for: Markerless Mouse Tracking for Social Experiments
Source: eNeuro. 2024 Feb 23;11(2):ENEURO.0154-22.2023. doi: 10.1523/ENEURO.0154-22.2023 (PMC10901195; doi:10.1523/ENEURO.0154-22.2023)

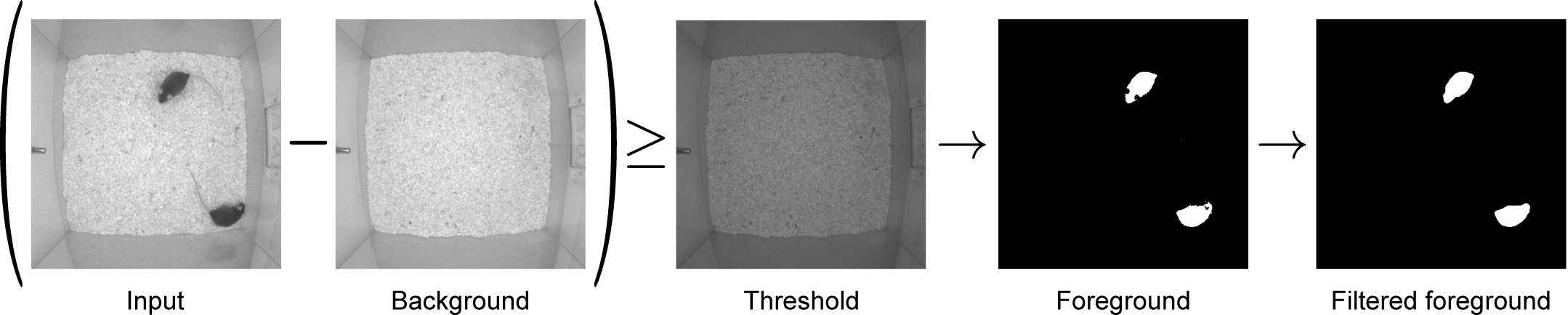

Supplement: Figure 2-1 — Foreground detection includes background subtraction, thresholding and a series of morphological operations: closing (9-pixel-radius circular structuring element) and opening (3-pixel radius circular structuring element). Download Figure 2-1, TIF file. [file eneuro-11-ENEURO.0154-22.2023-s009.tif]

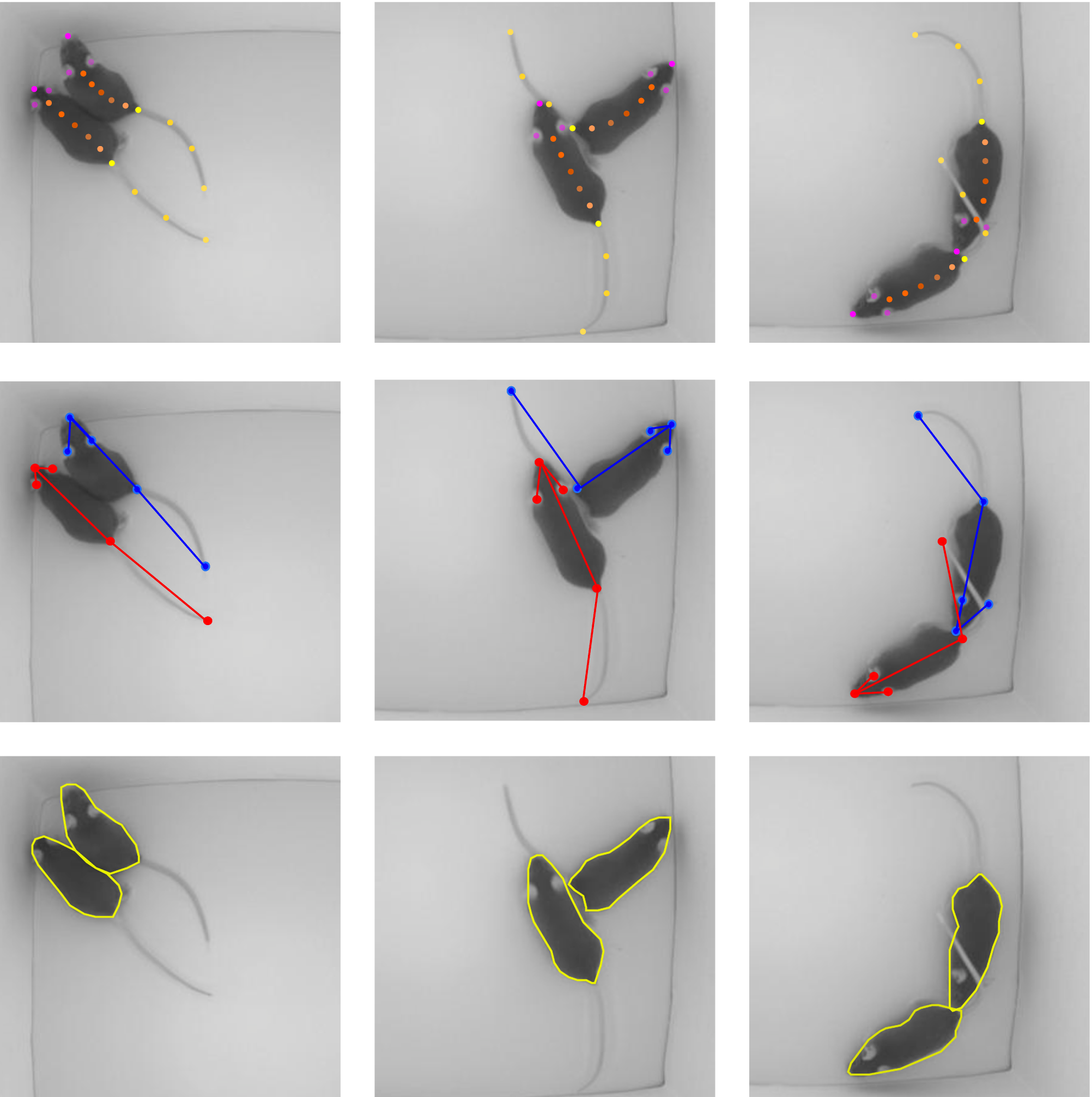

Supplement: Figure 3-1 — Example human annotations required for training DLC (top row), SLEAP (middle row), and our approach (bottom row). While annotation styles are different, human effort and time for both is comparable. Download Figure 3-1, TIF file. [file eneuro-11-ENEURO.0154-22.2023-s010.tif]

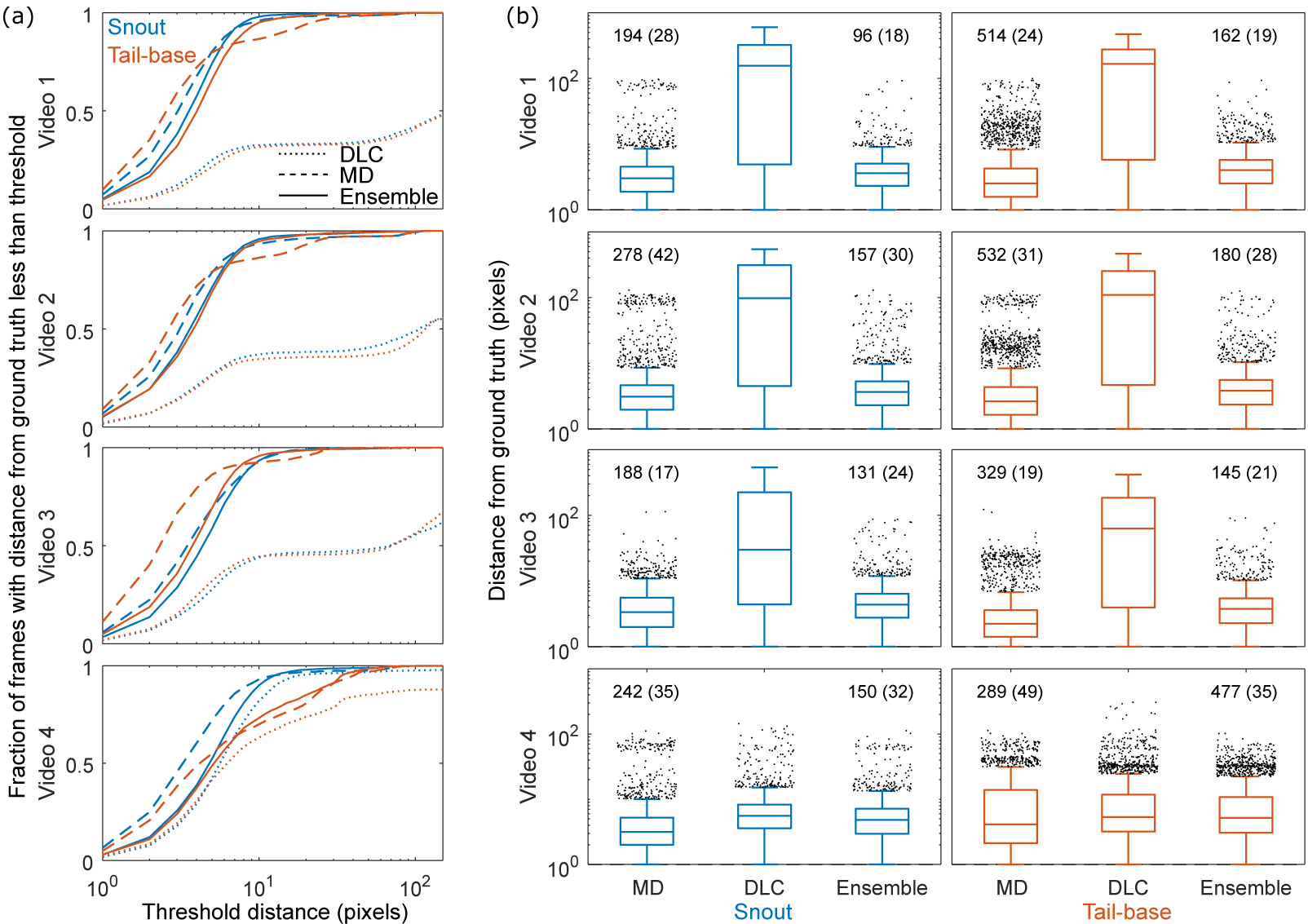

Supplement: Figure 6-1 — Performance across videos 1-4. (a) Fraction of frames with the mean distance between model predictions and human annotations below a varying threshold. (b) Boxplots showing errors in MD, DLC and Ensemble models. Plots show median, 25th and 75th percentile and outliers defined as > 75th percentile + 1.5 times the inter-quartile range. Text above outliers show number of outliers and average outlier within parenthesis. Download Figure 6-1, TIF file. [file eneuro-11-ENEURO.0154-22.2023-s011.tif]

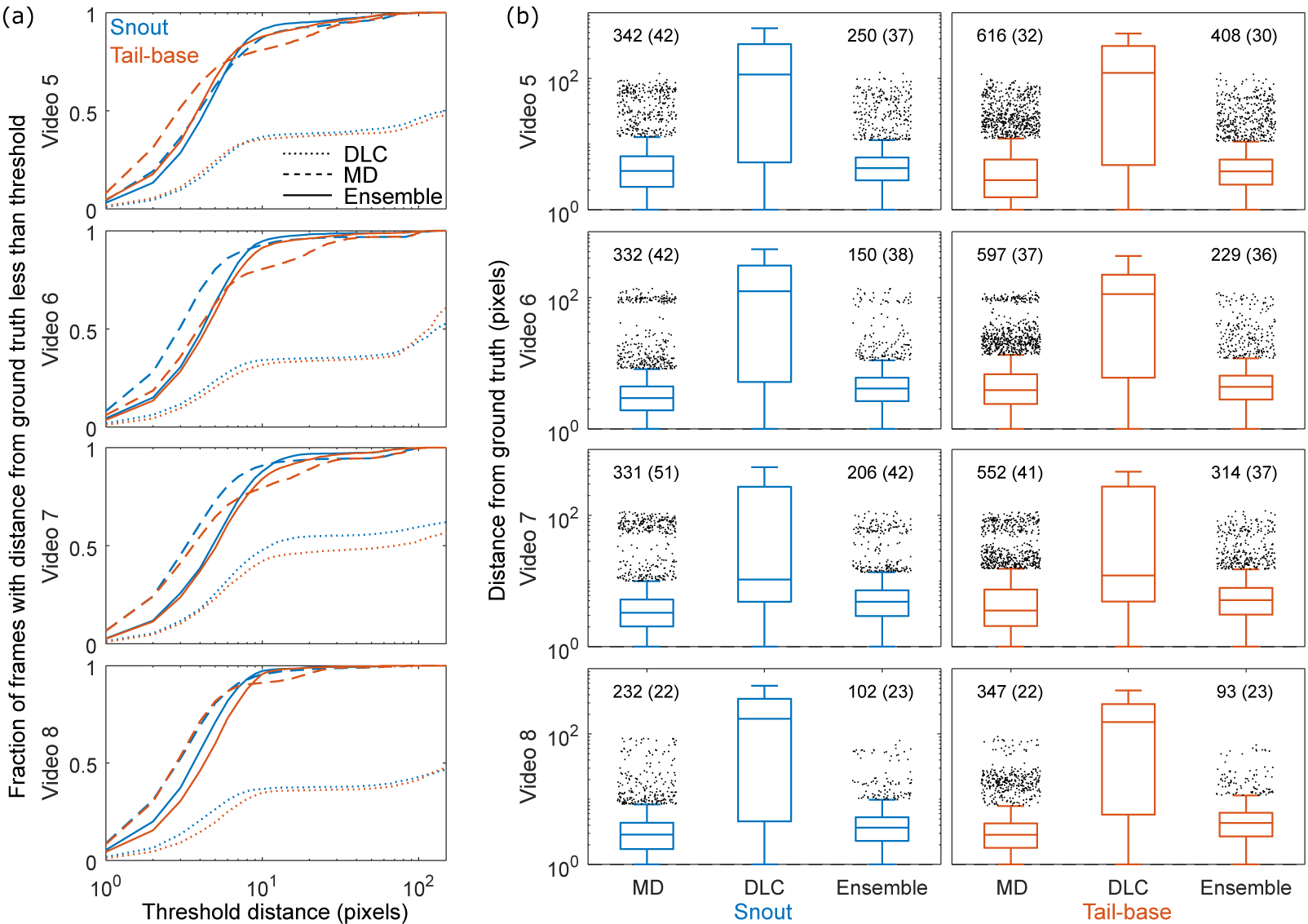

Supplement: Figure 6-2 — Performance across videos 5-8. (a) Fraction of frames with the mean distance between model predictions and human annotations below a varying threshold. (b) Boxplots showing errors in MD, DLC and Ensemble models. Plots show median, 25th and 75th percentile and outliers defined as > 75th percentile + 1.5 times the inter-quartile range. Text above outliers show number of outliers and average outlier within parenthesis. Download Figure 6-2, TIF file. [file eneuro-11-ENEURO.0154-22.2023-s012.tif]

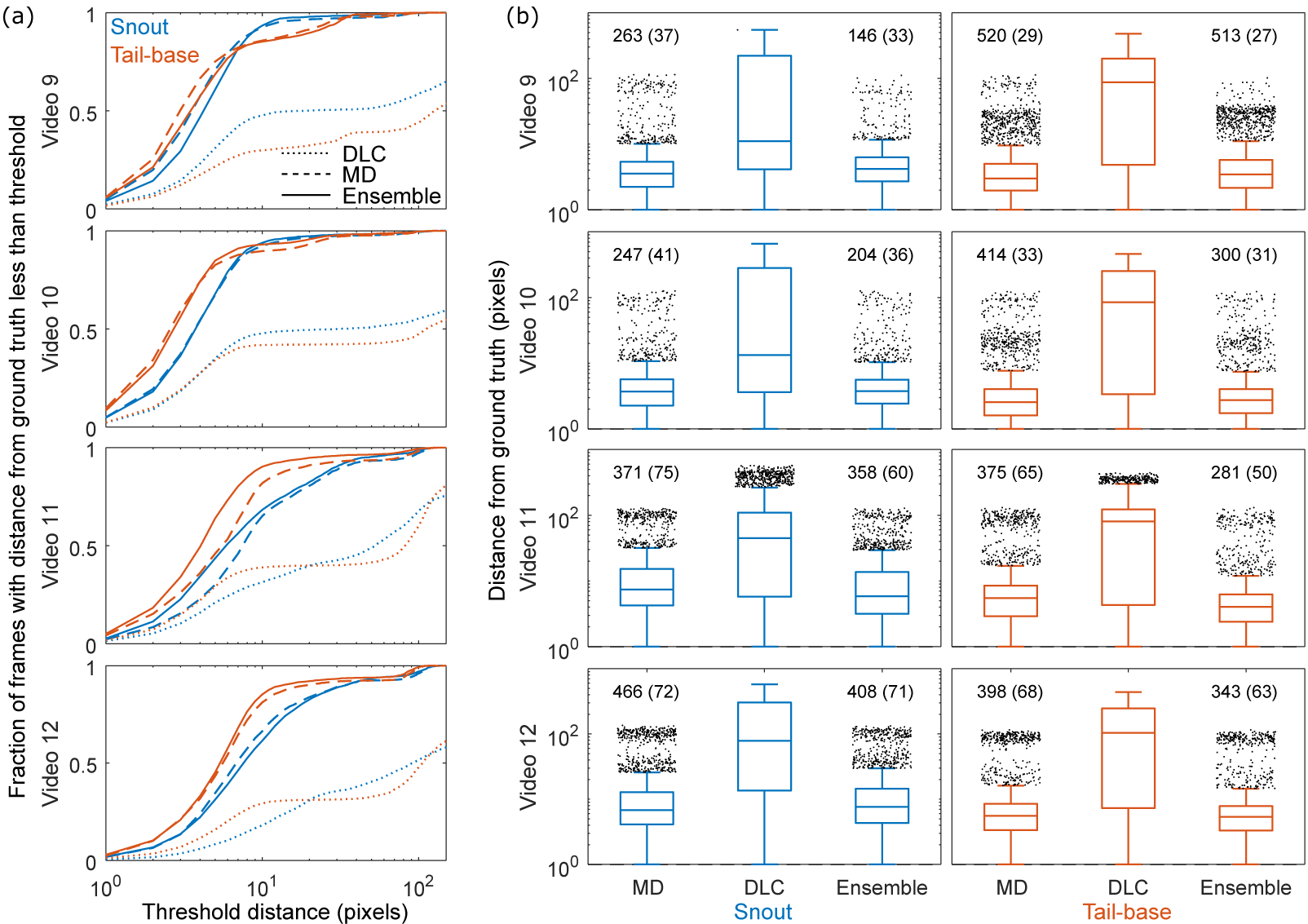

Supplement: Figure 6-3 — Performance across videos 9-12. (a) Fraction of frames with the mean distance between model predictions and human annotations below a varying threshold. (b) Boxplots showing errors in MD, DLC and Ensemble models. Plots show median, 25th and 75th percentile and outliers defined as > 75th percentile + 1.5 times the inter-quartile range. Text above outliers show number of outliers and average outlier within parenthesis. Download Figure 6-3, TIF file. [file eneuro-11-ENEURO.0154-22.2023-s013.tif]

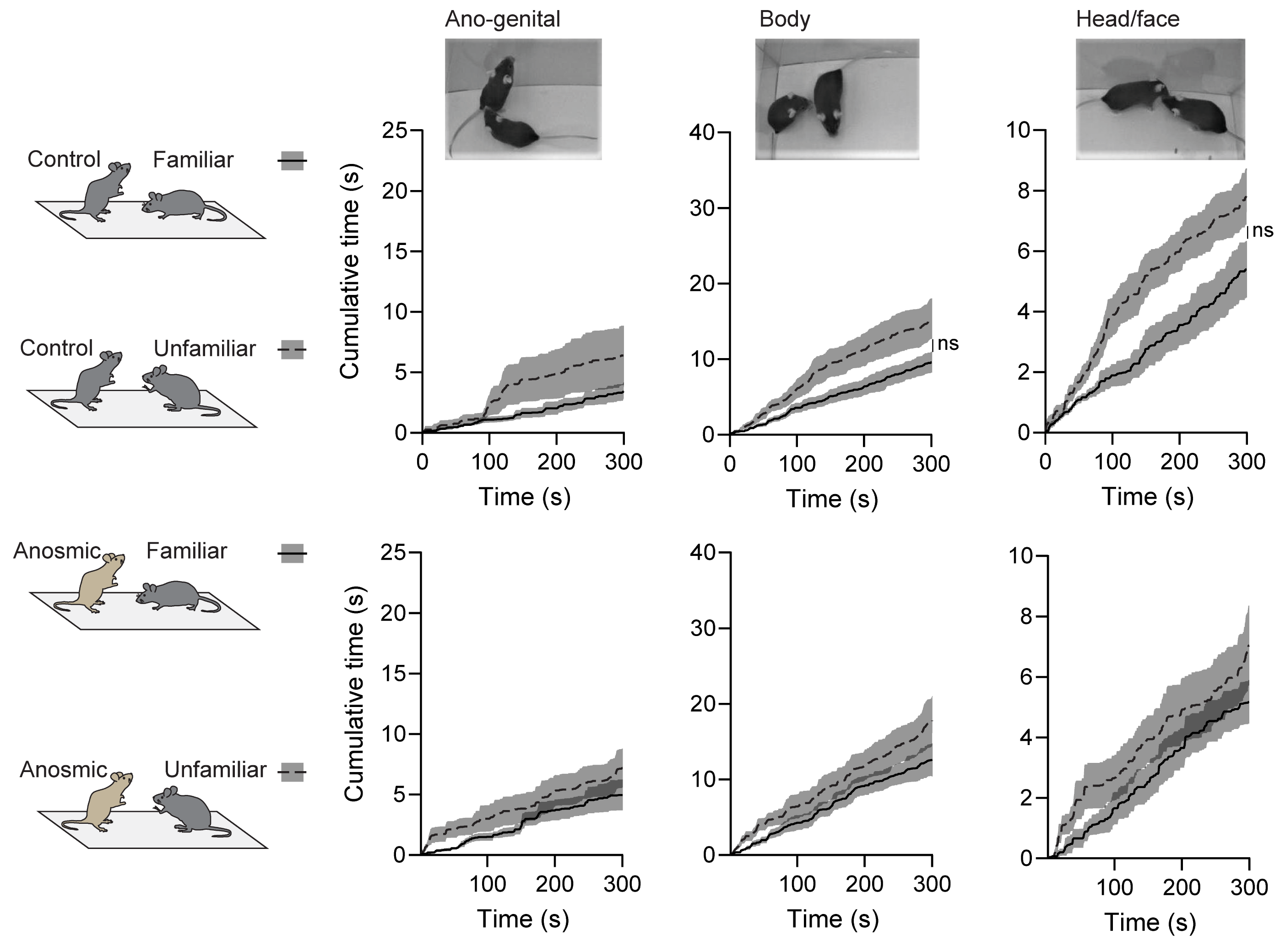

Supplement: Figure 8-1 — Social investigation behaviors of familiar or unfamiliar demonstrators towards control or anosmic observers. Social investigation behaviors include when the snout of the demonstrator was directed towards the ano-genital, body, or head/face region of the observer. Cumulative distributions show that familiar (solid line) and unfamiliar (dotted line) demonstrators spend similar amount of time engaged in each social investigation behavior when with a control (top) or anosmic (bottom) observer. Download Figure 8-1, TIF file. [file eneuro-11-ENEURO.0154-22.2023-s014.tif]
