# Supplementary material for: Markerless Mouse Tracking for Social Experiments
Source: eNeuro. 2024 Feb 23;11(2):ENEURO.0154-22.2023. doi: 10.1523/ENEURO.0154-22.2023 (PMC10901195; doi:10.1523/ENEURO.0154-22.2023)
Supplement: Table 10-1 — Velocities (calculated in 1 second bins as metres per second) correlated more frequently in control observer + demonstrator pairs (75%) than in anosmic observer + demonstrator pairs (25%). Download Table 10-1, DOCX file. [file eneuro-11-ENEURO.0154-22.2023-s015.docx]

| Treatment | Sex | Famil or Unfamil pair | Velocities correlate | r value | p value |
| --- | --- | --- | --- | --- | --- |
| Control | F | Famil | no | - | - |
| Control | F | Unfamil | yes | 0.1916 | 0.0009 |
| Control | F | Famil | no | - | - |
| Control | F | Unfamil | no | - | - |
| Control | F | Famil | yes | 0.2158 | 0.0002 |
| Control | F | Unfamil | yes | 0.1293 | 0.0254 |
| Control | F | Famil | yes | 0.411 | <0.0001 |
| Control | F | Unfamil | yes | 0.7067 | <0.0001 |
| Control | F | Famil | yes | 0.4741 | <0.0001 |
| Control | F | Unfamil | yes | 0.2865 | <0.0001 |
| Control | M | Famil | yes | 0.1969 | 0.0006 |
| Control | M | Unfamil | yes | 0.1492 | 0.0098 |
| Control | M | Famil | yes | 0.1699 | 0.0032 |
| Control | M | Unfamil | yes | 0.1848 | 0.0013 |
| Control | M | Famil | no | - | - |
| Control | M | Unfamil | yes | 0.3839 | <0.0001 |
| Anosmic | F | Famil | no | - | - |
| Anosmic | F | Unfamil | yes | 0.1207 | 0.0371 |
| Anosmic | F | Famil | no | - | - |
| Anosmic | F | Unfamil | no | - | - |
| Anosmic | F | Famil | yes | 0.4585 | <0.0001 |
| Anosmic | F | Unfamil | yes | 0.2173 | 0.0002 |
| Anosmic | M | Famil | no | - | - |
| Anosmic | M | Unfamil | no | - | - |
| Anosmic | M | Famil | no | - | - |
| Anosmic | M | Unfamil | no | - | - |
| Anosmic | M | Famil | no | - | - |
| Anosmic | M | Unfamil | no | - | - |
